# *ICE1* of *Poncirus trifoliata* functions in cold tolerance by modulating polyamine levels through interacting with arginine decarboxylase

**DOI:** 10.1093/jxb/erv138

**Published:** 2015-04-06

**Authors:** Xiao-San Huang, Qinghua Zhang, Dexin Zhu, Xingzheng Fu, Min Wang, Qian Zhang, Takaya Moriguchi, Ji-Hong Liu

**Affiliations:** ^1^Key Laboratory of Horticultural Plant Biology (MOE), College of Horticulture and Forestry Science, Huazhong Agricultural University, Wuhan 430070, PR China; ^2^National Institute of Fruit Tree Science, Tsukuba 305-8605, Japan

**Keywords:** bHLH, cold tolerance, polyamine, *Poncirus trifoliata* (L.) Raf., protein-interacting protein, ROS, transcription factor.

## Abstract

*ICE1* (*Inducer of CBF Expression 1*) encodes a MYC-like basic helix–loop–helix transcription factor that acts as a central regulator of cold response. In this study, we elucidated the function and underlying mechanisms of *PtrICE1* from trifoliate orange [*Poncirus trifoliata* (L.) Raf.]. *PtrICE1* was upregulated by cold, dehydration, and salt, with the greatest induction under cold conditions. PtrICE1 was localized in the nucleus and could bind to a MYC-recognizing sequence. Ectopic expression of *PtrICE1* in tobacco and lemon conferred enhanced tolerance to cold stresses at either chilling or freezing temperatures. Yeast two-hybrid screening revealed that 21 proteins belonged to the PtrICE1 interactome, in which PtADC (arginine decarboxylase) was confirmed as a *bona fide* protein interacting with PtrICE1. Transcript levels of *ADC* genes in the transgenic lines were slightly elevated under normal growth condition but substantially increased under cold conditions, consistent with changes in free polyamine levels. By contrast, accumulation of the reactive oxygen species, H_2_O_2_ and O_2_^–^, was appreciably alleviated in the transgenic lines under cold stress. Higher activities of antioxidant enzymes, such as superoxide dismutase and catalase, were detected in the transgenic lines under cold conditions. Taken together, these results demonstrated that PtrICE1 plays a positive role in cold tolerance, which may be due to modulation of polyamine levels through interacting with the *ADC* gene.

## Highlight

*ICE1* of *Poncirus trifoliata* (L.) Raf. plays a positive role in cold tolerance and modulates polyamine synthesis by interacting with arginine decarboxylase.

## Introduction

Cold is one of the most devastating abiotic stresses that impair plant growth and development, reduce productivity, and limit geographical distribution of natural populations. As most citrus commercial cultivars are not cold hardy, cold has been documented to cause great economic loss in several important citrus-producing regions globally (Sahin-Çevik and Moore, 2006). Therefore, improvement of cold tolerance is a major task of citrus breeding programmes. However, due to special reproductive natures, such as polyembryony, a long juvenile period, and a high degree of heterozygosity (Talon and Gmitter, 2008), limited achievements have been made so far to improve citrus cold tolerance by means of traditional breeding approaches. A vast number of elegant studies have provided evidence showing that genetic engineering is a powerful strategy for creating germplasms with enhanced cold tolerance (Lata and Prasad, 2011; Krasensky and Jonak, 2012). The underlying prerequisite for this strategy is to characterize valuable genes that can be genetically engineered.

Being sessile organisms, plants have evolved to interpret and respond to adverse environmental cues by molecular, physiological, and biochemical alterations, enabling them to survive under harsh situations (Nakashima *et al.*, 2009; Thomashow, 2010; Theocharis *et al.*, 2012). Over the last decades, enormous progress has been made in deciphering significant components implicated in the cold signalling network (Wisniewski *et al.*, 2014; Shi *et al.*, 2015). One of the most important milestones has been the identification of C-repeat-binding factor (*CBF*) genes, including *CBF1*, *CBF2*, and *CBF3* (Liu *et al.*, 1998; Thomashow 2001; Chinnusamy *et al.*, 2006; Medina *et al.*, 2011). The *CBF*s, conserved among monocots and dicots, play crucial roles in regulating a large spectrum of cold-regulated (COR) genes, collectively called the CBF regulon, implying that they act as central hub in the cold signalling network (Yamaguchi-Shinozaki and Shinozaki, 2005; Thomashow, 2010; J. H. Liu *et al.*, 2014). Another important breakthrough has been characterization of *Inducer of CBF Expression 1* in *Arabidopsis thaliana* (*AtICE1*).

*ICE1* encodes a MYC-type basic helix–loop–helix (bHLH) transcription factor (Chinnusamy *et al.*, 2003). Overexpression of *ICE1* led to stronger induction of *CBF3* and downstream *COR* genes, but induction of these genes was impaired in an *ice1* mutant. Further in-depth work showed that ICE1 regulated the expression of *CBF3* by binding to a *cis*-acting element containing the MYC-recognizing (MYCR) core sequence (CANNTG) in the *CBF3* promoter region (Chinnusamy *et al.*, 2003; Lee *et al.*, 2005). In addition, ICE2, an ICE1 homologue in *Arabidopsis thaliana*, was identified as functioning in regulating cold tolerance via modulation of *CBF1* expression (Fursova *et al.*, 2009). These findings suggest that ICE1 or ICE2 acts as a master regulator of the cold signalling pathway and plays a pivotal role in mediating plant responses to cold, thus establishing ICE1–CBF–COR as the most significant signalling cascade. To date, *ICE1* and *ICE2* homologues have been unravelled in various plants, such as *Calellia sinensis* (Wang *et al.*, 2012), apple (Feng *et al.*, 2012), tomato (Feng *et al.*, 2013), *Chrysanthemum dichrum* (Chen *et al.*, 2013), trifoliate orange (Huang *et al.*, 2013), *Phalaenopsis aphrodite* (Peng *et al.*, 2014), and banana (Shan *et al.*, 2014). Recently, Hu *et al.* (2013) reported that jasmonate functions positively as a critical upstream signal regulating the ICE1-mediated cold response, which adds new vision to our understanding of the cold stress response.

It is generally accepted that ICE1 functions in cold tolerance due to its essential role in governing the expression of CBFs, which in turn regulate the downstream target genes (Chinnusamy *et al.*, 2003; Van Buskirk and Thomashow, 2006; Thomashow, 2010). However, the possibility of the existence of other unexplored mechanisms underlying the role of ICE1 cannot be fully ruled out. As a matter of fact, recent work by Chen *et al.* (2013) demonstrated that, in addition to the established ICE1–CBF cascade, CdICE1 of *Chrysanthemum dichrum* could mediate freezing tolerance by regulating expression of the microRNA miR398, which in turn modified transcript levels of superoxide dismutase (*SOD*) associated with reactive oxygen species (ROS) scavenging. Therefore, it is reasonable to hypothesize that some undetermined molecular mechanisms might also contribute to the cold tolerance imparted by ICE1 and its homologues.

Trifoliate orange [*Poncirus trifoliata* (L.) Raf.] has deciduous leaves and undergoes winter dormancy. It is widely used as a citrus rootstock to boost cold tolerance in citrus cultivars grafted onto it due to its striking tolerance to extremely low temperatures after thorough cold acclimation. This unusual property makes trifoliate orange a major source of candidate genes for cold tolerance improvement in citrus. In this study, we report that overexpression of *P. trifoliata ICE1* (*PtrICE1*) led to enhanced cold tolerance in tobacco and lemon transgenic lines. Yeast two-hybrid (Y2H) screening gave rise to 21 potential PtrICE1-interacting proteins, in which *P. trifoliata* arginine decarboxylase (PtADC) was experimentally confirmed. We also demonstrated that transcript levels of *ADC* genes were elevated in the transgenic plants of tobacco and lemon, in particular under cold treatment, concomitant with an increase in free polyamine levels compared with the wild type (WT). All of these findings indicate that PtrICE1 is involved in the regulation of cold tolerance via modulating polyamine synthesis by interacting with ADC.

## Materials and methods

### Plant materials and treatments

Young shoots collected from 2-year-old trifoliate orange plants grown in a greenhouse under natural illumination were used to analyse expression of *PtrICE1*. The shoots were first incubated in distilled water for 48h at room temperature before being treated with various abiotic stresses, including dehydration, cold, and salt. For dehydration treatment, the shoots were placed in empty flasks for 6h; leaves were collected at 0, 0.5, 1, 3, and 6h after dehydration treatment. For cold stress, the shoots were kept in a growth chamber set at 4 °C for 72h; the leaves were harvested at 0, 5, 24, 48, and 72h after initiation of the treatment. For salt stress, the shoots were inserted into flasks containing 200mM NaCl; the leaves were collected at the same time points as for cold treatment. The leaf samples were frozen immediately in liquid nitrogen and stored at –80 °C until use for expression analysis.

### Isolation and bioinformatics analysis of PtrICE1

A database search against Harvest (http://harvest.ucr.edu) was carried out using ‘ICE1’ as an entry keyword, as performed previously (Huang *et al.*, 2013). Sequence analysis of the outputs showed that one (782bp) was annotated as an ICE1 homologue. As the 5’ terminus of this sequence was missing, 5’ rapid amplification of cDNA ends (5’-RACE) PCR with a gene-specific primer (GSP, 5’-CTGATCTCCACCAGTGATAGTGCT-3’) was performed using a SMART RACE cDNA Amplification kit (Clontech, USA) based on the manufacturer’s instructions. The 5’-RACE sequence and the initial one were combined into a contig containing an open reading frame (ORF), which was validated by reverse transcription (RT)-PCR with a pair of primers (GSP1; see Supplementary Table S1 at *JXB* online, in which primers used for this study are listed unless otherwise stated). The sequence was subjected to bioinformatics analysis, as described by Liu *et al.* (2009). In addition, a phylogenetic tree was reconstructed by the neighbour-joining method using MEGA 4.0.

### Subcellular localization of PtrICE1

The full-length cDNA of *PtrICE1* without a stop codon was amplified by RT-PCR with primer pair GSP2 (Supplementary Table S1), and fused to the N terminus of the green fluorescent protein (GFP) gene of the pBI121 vector, driven by the cauliflower mosaic virus (CaMV) 35S promoter. The subcellular localization of PtrICE1 was examined by *Agrobacterium tumefaciens*-mediated transient expression of the fusion vector (PtrICE1–GFP) and control vector (GFP) in onion epidermal cells (Huang *et al.*, 2011). The transformed cells were cultured in the dark on MS medium for 1–2 d before visual observation under a universal fluorescence microscope (BX61; Olympus, Tokyo, Japan).

### Transcriptional activation and MYCR-binding assay of PtrICE1

The ORF of *PtrICE1* generated by RT-PCR using primer pair GSP3 (Supplementary Table S1) was digested with *Bam*HI and *Xho*I and inserted into pENTR3C (Invitrogen). The recombinant vector (pENTR3C-PtrICE1) was fused in frame downstream of the yeast GAL4 DNA-binding domain (BD) of vector pDEST32 (Invitrogen). The fusion vector and negative control (pDEST32) were transformed independently into yeast MaV203 strain (Invitrogen) according to the manufacturer’s instructions. The transformed yeast cells were plated for 3 d on SD/–Leu/–Trp or SD/–Leu/–Trp/–His medium added with or without 5 and 15mM 3-aminotriazole (3-AT), followed by growth observation.

A yeast one-hybrid assay (Clontech) was performed to investigate whether PtrICE1 could bind to the MYCR sequence. The PtrICE1 ORF was amplified using the primer pair GSP4 (Supplementary Table S1) and fused to the GAL4 activation domain (AD) in the pGADT7 vector to create pGADT7-PtrICE1. A 66bp DNA fragment composed of triple tandem repeats of a sequence containing MYCR (CACATG) was inserted into the pHIS2 vector, generating a recombinant construct of pHIS2-MYCR. Thereafter, pGADT7-PtrICE1 and pHIS2-MYCR were co-transformed into yeast cells (strain Y187), which were grown for 3 d on SD/–Leu/–Trp/–His medium with or without 15mM 3-AT.

### Expression analysis by quantitative real-time RT-PCR (qPCR)

Total RNA was isolated using Trizol reagent (Invitrogen) and digested with RNase-free DNaseI (TaKaRa) to remove contaminating DNA. About 1 μg of total RNA was used to synthesize the first-strand cDNA with Moloney murine leukemia virus reverse transcriptase (Promega) in a 20 µl reaction mixture. To investigate gene expression (*PtrICE1* and *ADC*), qPCR analysis with a SYBR Green PCR kit (SYBR Green; Applied Biosystems) was carried out in an ABI 7500 Real-Time System (PE Applied Biosystems, Foster City, CA, USA). The PCR mixture (10 µl) contained 5 µl of 2× SYBR Green RealMasterMix, 50ng of cDNA, and 0.2 µM of each primer (GSP5 pair; Supplementary Table S1). Thermal conditions were 50 °C for 2min and 95 °C for 10min, followed by 40 cycles of 95 °C for 15 s and 60 °C for 1min. Each sample was analysed in four replicates, and the 2^–ΔΔCt^ method was applied to calculate the relative expression levels of each gene. *Actin* and *Ubiquitin* were analysed in parallel as internal reference controls for trifoliate orange/lemon and tobacco, respectively, to normalize expression levels.

### Transformation and characterization of transgenic plants

The full-length *PtrICE1* gene was inserted into binary vector pBI121 linearized with *Xho*I and *Kpn*I, under the control of the *CaMV 35S* promoter. The recombinant plasmid was introduced into *Agrobacterium tumefaciens* strain EHA105 by heat shock. *Agrobacterium*-mediated transformation of tobacco (*Nicotiana tabacum*) and lemon (*Citrus limon*) was carried out (Horsch *et al.*, 1985; Huang *et al.*, 2010; Fu *et al.*, 2011). Kanamycin-resistant plants derived from independent transformation events were verified by genomic PCR using two pairs of primers (NPTII and CaMV 35S-PtrICE1; Supplementary Table S1). In addition, overexpression of *PtrICE1* was assayed by semi-quantitative PCR or qPCR in transgenic lines of lemon and tobacco, respectively. Homozygous tobacco plants at the T_2_ generation and vegetatively multiplied lemon plants were used for a stress tolerance assay and physiological measurement.

### Assessment of cold tolerance in transgenic lines

Tobacco plants were treated with cold at either chilling (4 °C) or freezing (0 °C) temperatures. For chilling treatment, tobacco seedlings planted at 25 °C were kept at 4 °C for 48h or 5 d. Electrolyte leakage and ROS levels were assessed after the chilling treatment. In another experiment, 35-d-old plants were first treated at 0 °C for 24h and then moved to an ambient environment for further growth for 5 d (recovery), followed by assessment of survival rate. Leaves collected before and after the freezing treatment were used for analysis of polyamine levels, ROS accumulation, and expression of *ADC* (*NtADC1* and *NtADC2*) genes.

For lemon, two transgenic lines (#17 and #21) and WT plants were treated for 4h at –3 °C and then moved to an ambient environment for further growth for 5 d (recovery). The leaves were sampled before and/or after the freezing treatment for analysis of electrolyte leakage, chlorophyll content, cell death, ROS accumulation, polyamine levels, and *ADC* gene (*ClADC*) expression. In addition, they were treated at –6 ºC for 1.5h, followed by recovery for 4 d to further compare the freezing tolerance.

### Physiological measurements and histochemical staining

Electrolyte leakage was measured according to previous studies (Huang *et al.*, 2010; Wang *et al.*, 2011). Chlorophyll contents [chlorophyll *a* (Ca); chlorophyll *b* (Cb), and total chlorophyll (Ct)] were quantitatively measured based on the method of Liu *et al.* (2008). Histochemical staining with 3,3’-diaminobenzidine (DAB) and nitroblue tetrazolium (NBT) was used to analyse the *in situ* accumulation of H_2_O_2_ and O_2_^–^, respectively, as depicted by Huang *et al.* (2010) and Shi *et al.* (2010). Quantitative measurement of H_2_O_2_ and O_2_^–^ was performed using specific detection kits (Nanjing Jiancheng Bioengineering Institute, China), and protein concentration was determined colorimetrically (Bradford, 1976). Cell death was examined with trypan blue staining based on the method of Pogány *et al.* (2009).

### Quantification of polyamine levels by high-performance liquid chromatography (HPLC)

Free polyamines were extracted with 5% cold perchloric acid containing 500mg l^–1^ of dithiothreitol as described by Liu and Moriguchi (2007) and then derivatized with benzoyl chloride. Polyamines were separated at room temperature using an Agilent HPLC system (USA) equipped with a C_18_ reversed-phase column (4.6×150mm, particle size 5 μm) and UV detector (230nm), using hexamethylene diamine as an internal control. The solvent (HPLC-grade methanol and water) changed from 50:50 (v/v) to 95:5 in 10min at a flow rate of 0.7ml min^–1^. Quantification of the polyamine levels, expressed as nmol g^–1^ of fresh weight, was performed in triplicate.

### Y2H-based screening of PtrICE1-interacting proteins

Full-length and truncated *PtrICE1* (deletion of the transactivation region at aa 49–98) were amplified with primer pairs GSP3 and GSP6 (Supplementary Table S1), respectively, and inserted into pENTRTM3C (Invitrogen). The recombinant constructs were then fused in frame with the GAL4 BD of pDEST32 vector using a homologous recombination system (ProQuest Two-Hybrid System; Invitrogen). The fused proteins were expressed in yeast strain MaV203 cells to examine autoactivation. For Y2H analysis, a cDNA library was constructed with trifoliate orange treated at 4 °C for 72h, and then screened using the truncated PtrICE1 as bait following the manufacturer’s instructions (Invitrogen). The yeast cells were cultured for 7 d at 30 °C on selection medium (SD/–Leu/–Trp/–His) supplemented with 50mM 3-AT. Positive clones were sequenced and analysed bioinformatically.

### Protein–protein interaction analysis

To confirm an interaction between PtrICE1 and PtADC, full-length cDNA of *PtADC* was amplified using primer pair GSP7 (Supplementary Table S1) and cloned into the *Xho*I and *Kpn*I sites of pDEST22 vector to get AD–PtADC (prey), while the truncated PtrICE1 was inserted into *Xho*I and *Kpn*I sites of pDEST32 vector to generate BD–PtrICE1 (bait). The two constructs were co-transformed into yeast cells, which were cultured as described above. For bimolecular fluorescence complementation (BiFC) analysis (Walter *et al.*, 2004), the *PtrICE1* ORF without a stop codon was PCR amplified with primer pair GSP9 (Supplementary Table S1) and then subcloned into pSPYNE-35S containing the N-terminal fragment of yellow fluorescent protein (nYFP) to get PtrICE1–nYFP. Meanwhile, full-length *PtADC* without a stop codon was amplified using primer pair GSP10 (Supplementary Table S1) and inserted into pSPYCE-35S containing the C-terminal fragment of YFP (cYFP) to generate PtrADC–cYFP. The recombinant vectors were introduced into *Agrobacterium tumefaciens* strain EHA105 and used for transient expression in tobacco (*Nicotiana benthamiana*) via leaf infiltration (Walter *et al.*, 2004). YFP fluorescence in the epidermis was monitored via a universal fluorescence microscope (ECLIPSE 90i). Positive (AtbZIP63–nYFP and AtbZIP63–cYFP) and negative (PtrICE1–nYFP and cYFP) controls were processed in the same way.

### Statistical analysis

The data were statistically processed using the SAS software package (SAS Institute); statistic difference was compared using one-way analysis of variance based on a *t*-test, at the significance levels of *P*<0.05, *P*<0.01, and *P*<0.001.

## Results

### Cloning and bioinformatics analysis of PtrICE1

**Fig. 1.**
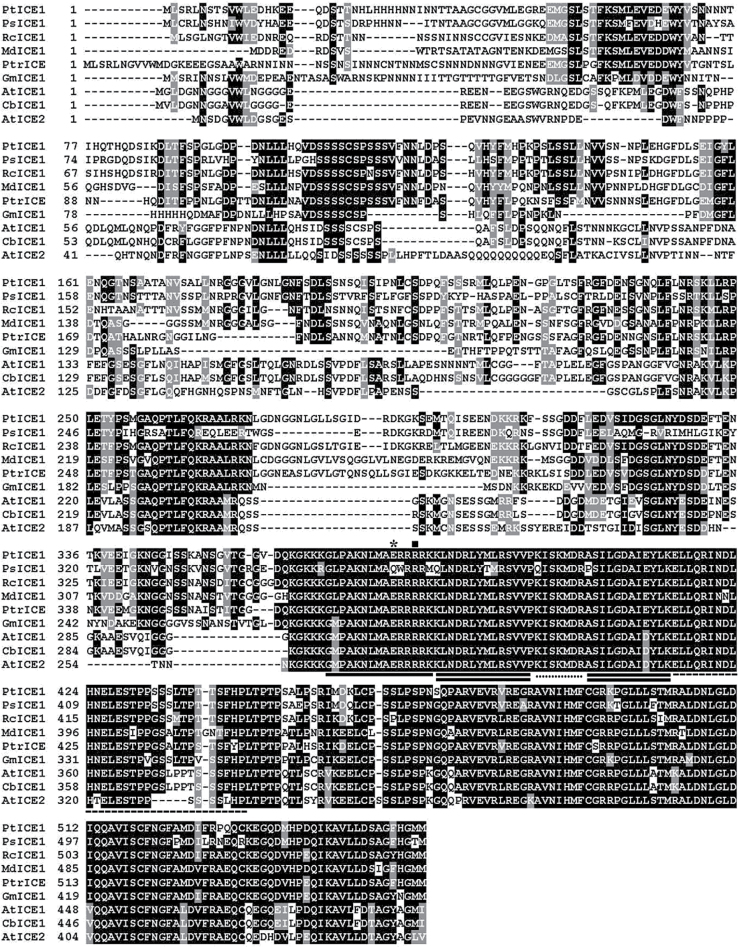
Amino acid sequence alignment of PtrICE1 and ICE1 or ICE2 of other plants, comprising *Populus trichocarpa* (NCBI Protein no. ABN58427), PsICE1 of *Populus suaveolens* (ABF48720), RcICE1 of *Ricinus communis* (EEF51703), MdICE1 of apple (ABS50251), GmICE1 of soybean (ACJ39211), AtICE1 and AtICE2 of *Arabidopsis thaliana* (AAP14668 and BAC42644, respectively), and CbICE1 of *Capsella bursa-pastoris* (AAS79350). Identical and similar residues are shown in black and grey background, respectively. The single bold line below the sequence indicates the basic region, while double lines represent the helix regions, which are connected by a loop, indicated by the dotted line. The dashed line shows the leucine-zipper region. The asterisk and square indicate glutamate and arginine, respectively.

To obtain the trifoliate orange *PtrICE1* gene, we first searched the Harvest database using ICE1 as an entry, which yielded different outputs (Huang *et al.*, 2013), including an expressed sequence tag (EST) of 782bp. Homology comparison in the NCBI database showed that the EST exhibited high sequence homology to ICE1. As the 5’ terminus was missing, 5’-RACE PCR was performed to obtain the 5’-terminus sequence, leading to amplification of a sequence of 1246bp. Merging of the 5’-RACE sequence and the original EST generated a sequence of 1869bp. Analysis in ORF Finder (http://www.ncbi.nlm.nih.gov/gorf/gorf.html) indicated that it contained a complete ORF of 1680bp, along with a 160bp 5’-untranslated region and a 29bp 3’-untranslated region. For convenience of description, the full-length cDNA was designated *PtrICE1* and has been deposited in GenBank under accession number KJ812152. PtrICE1 was predicted to encode a protein of 559 aa, with an estimated molecular mass of 61.2kDa and a *p*I of 5.27. Multiple alignments indicated that PtrICE1 shared a high degree of sequence identity with ICE1 proteins of eight other plants at the C terminus, whereas they varied extensively among each other at the N terminus (Fig. 1). Motif scanning revealed that PtrICE1 contained a MYC-like bHLH domain of 48 aa (from aa 371 to 418) composed of a 15 aa basic region and two helices (14 aa each) that were connected by a 5 aa loop. In addition, an acidic region existed at the N terminus (aa 49–98) of PtrICE1. An unrooted phylogenetic tree was reconstructed using complete amino acid sequences of PtrICE1 and ICE1s of other plants, in which PtrICE1 was most closely related to RcICE1 of *Ricinus communis* (Supplementary Fig. S1 at *JXB* online). In addition, at the amino acid level, PtrICE1 displayed 46.4% sequence identity to PtrbHLH, an ICE1-like bHLH protein in *P. trifoliata* (Huang *et al.*, 2013; Supplementary Fig. S2 at *JXB* online).

### PtrICE1 is localized in the nucleus and has transactivation activity in yeast

**Fig. 2.**
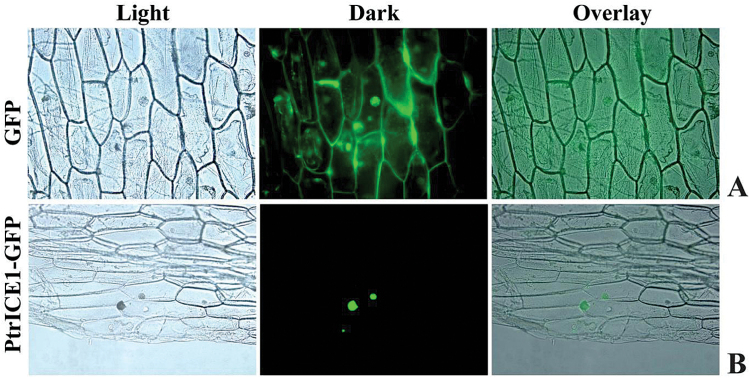
Nuclear localization of PtrICE1. Onion epidermis was transformed with vectors containing GFP (A), used as a control, or PtrICE1–GFP fusion protein (B). Subcellular localization was visualized by fluorescence microscopy. Representative images show cells expressing GFP or PtrICE1–GFP fusion protein under bright field (light) or UV field (dark), together with corresponding overlaid images.

Sequence analysis showed that PtrICE1 contained two nuclear localization signals (aa 298–312 and 366–383), implying that it may be localized in the nucleus. To verify this, the subcellular localization pattern of PtrICE1 was examined using a fusion protein of PtrICE1 and GFP, under the control of the CaMV 35S promoter. Microscopic visualization showed that the control GFP was uniformly distributed throughout the whole cell (Fig. 2A), while the PtrICE1–GFP fusion protein was observed exclusively in the nucleus (Fig. 2B), indicating that PtrICE1 was a nuclear protein.

**Fig. 3.**
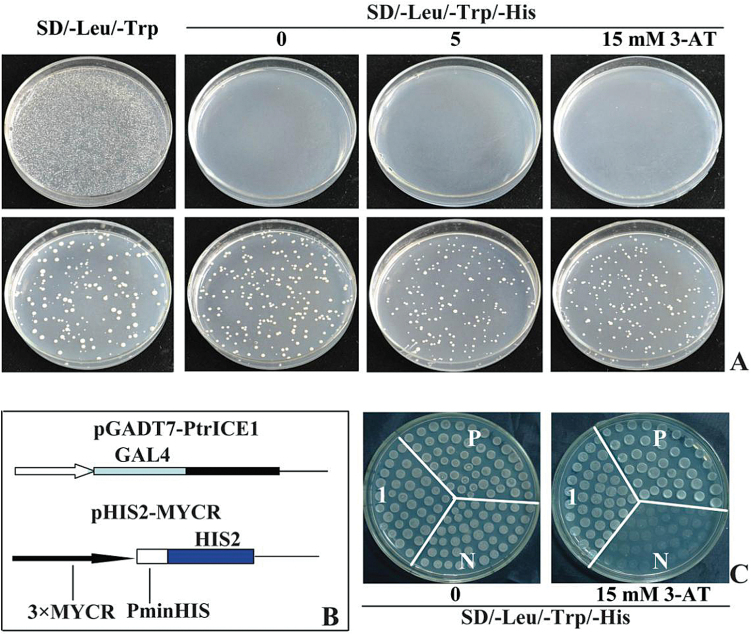
Transcriptional activation assay of PtrICE1. (A) Growth of yeast cells (strain MaV203) transformed with either control vector (upper panels) or fusion vector harbouring PtrICE1 (bottom panels) on SD/–Leu/–Trp or SD/–Leu/–Trp/–His with or without 3-AT. (B) Schematic illustration of the vectors (pGADT7-PtrICE1 and pHIS2-MYCR) used for the transactivation assay. (C) Growth of yeast cells co-transformed with vectors of the positive control (P), negative control (N), and pGADT7-PtrICE1 with pHIS2-MYCR (1) on SD/–Leu/–Trp/–His with or without 15mM 3-AT.

In addition, the Y2H system was used to investigate whether PtrICE1 functioned as a transcriptional activator. The yeast cells carrying the control plasmid grew well on SD/-Leu/-Trp medium, but failed to grow on SD/-Leu/-Trp/-His medium added with or without 3-AT. By contrast, the yeast cells transformed with the fusion plasmid containing PtrICE1 grew healthily on all tested media (Fig. 3A), indicating that PtrICE1 had transcriptional activation activity.

ICE1 of *Arabidopsis thaliana* can bind to the MYCR *cis*-acting element (Chinnusamy *et al.*, 2003), which compelled us to examine whether PtrICE1 could also bind to a sequence containing the MYCR sequence. For this purpose, the *PtrICE1* ORF was fused to the GAL4 AD in the pGADT7 vector to generate an effector, while a reporter construct (pHIS2-MYCR) was created by ligating a sequence containing three repeats of MYCR in the pHIS2 vector. The yeast (strain Y187) cells co-transformed with the effector and reporter grew normally, whereas those of control cells died on the selection medium (Fig. 3B), suggesting that PtrICE1 binds to MYCR and activates the reporter genes in yeast.

### Expression patterns of PtrICE1 under various treatments

**Fig. 4.**
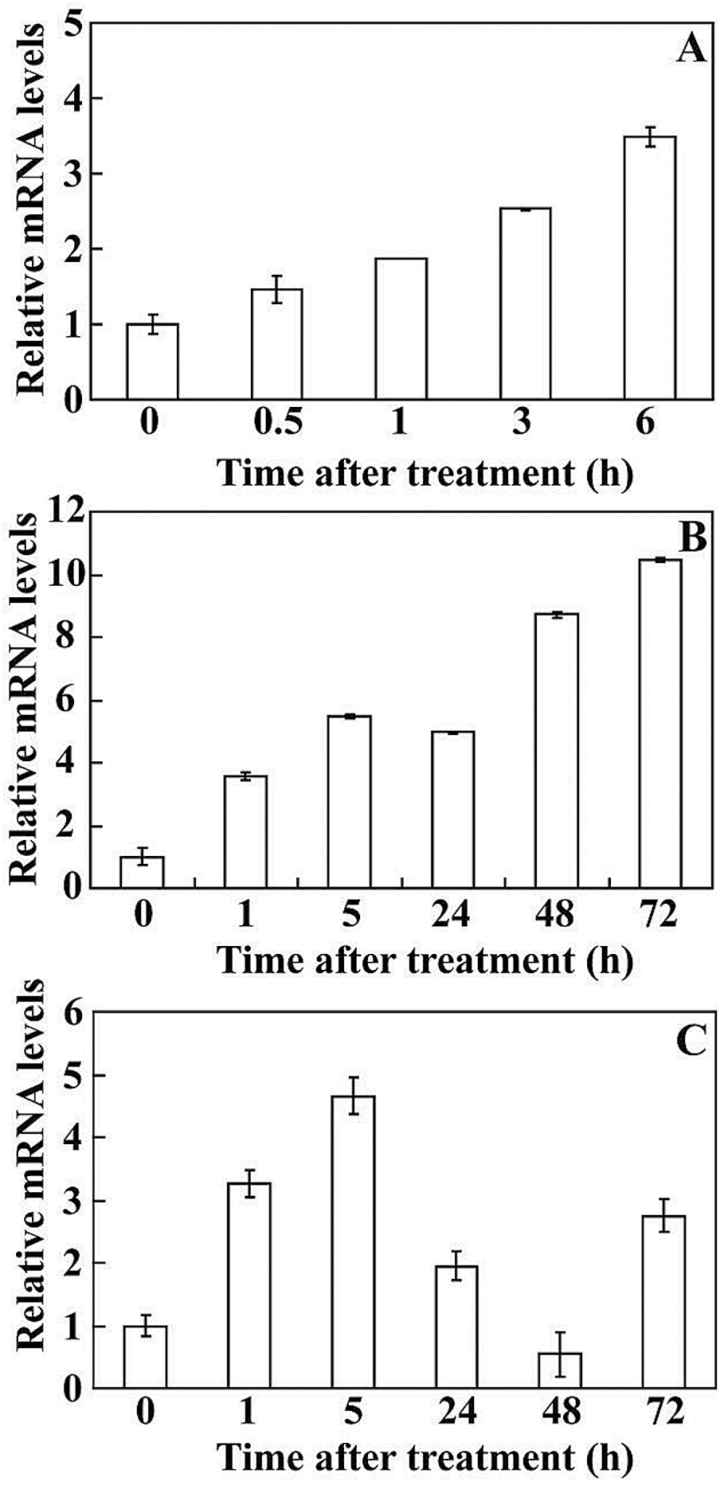
Expression of *PtrICE1* under abiotic stresses, analysed by qPCR. Time-course expression patterns of *PtrICE1* in response to dehydration (A), cold (B), and salt stress (C). Error bars show the standard deviation based on four replicates.

In order to illustrate the potential function of *PtrICE1*, expression patterns of *PtrICE1* under various abiotic stresses were analysed by qPCR. *PtrICE1* mRNA accumulated at 0.5h after dehydration, and continued to increase, reaching a maximum at 6h (Fig. 4A). Under cold treatment, the transcript level of *PtrICE1* was pronouncedly induced by nearly 4 folds, and then increased progressively to its highest level at 72h (more than 10-fold the initial level) (Fig. 4B). *PtrICE1* transcripts exhibited steady elevation within 5h of salt stress initiation, but gradually decreased to their lowest value at 48h, followed by an increase at the last time point (Fig. 4C).

### Overexpression of *PtrICE1* confers cold tolerance in transgenic tobacco and lemon

**Fig. 5.**
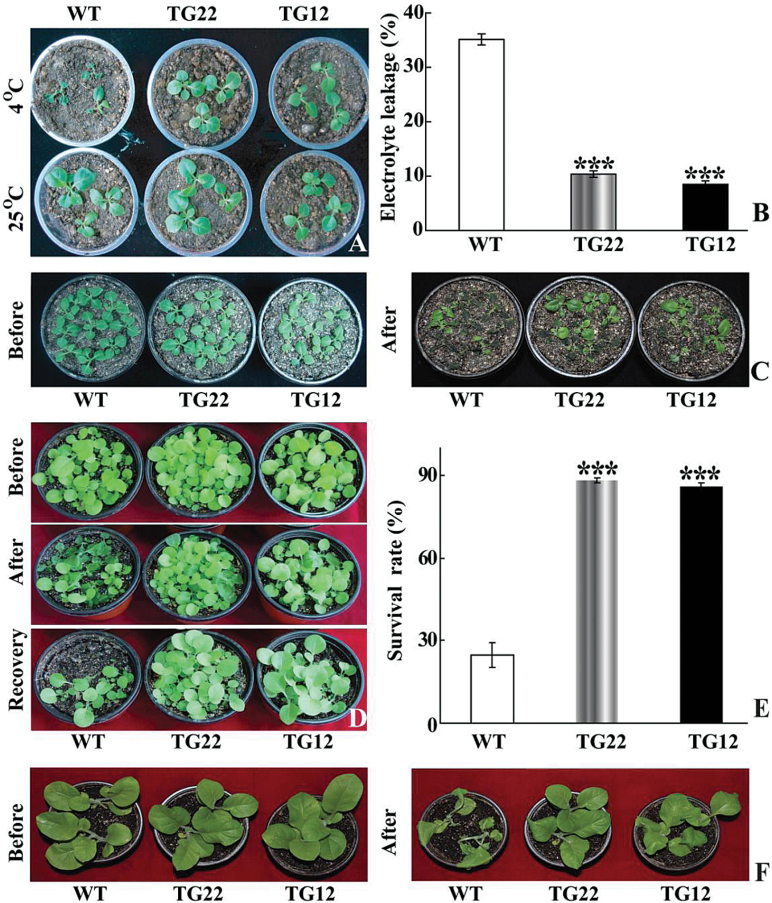
Overexpression of *PtrICE1* confers enhanced cold tolerance in tobacco. (A) Plant phenotype of tobacco WT and transgenic (TG22 and TG12) plants before and after cold treatment for 2 d at 4 °C. (B) Electrolyte leakage of WT and transgenic plants after cold treatment. (C) Plant phenotypes of WT and transgenic lines before and after cold treatment at 4 °C for 5 d. (D) Plant phenotypes of WT and transgenic lines before and after cold treatment at 0 °C for 1 d, followed by recovery growth for 5 d in an ambient environment. (F) Survival rates of WT and transgenic plants. (G) Plant phenotype of larger WT and transgenic plants before and after cold treatment at 4 °C for 1 d. Asterisks indicate that the transgenic lines and WT are significantly different from each other (***P<0.01). (This figure is available in colour at *JXB* online.)

As *PtrICE1* transcripts were induced to their highest level by cold, efforts were then made to illustrate the role of this gene in cold tolerance. To this end, we first generated transgenic tobacco plants overexpressing *PtrICE1* via *Agrobacterium*-mediated transformation. Two independent lines (hereafter designated as TG22 and TG12) with higher transcript levels of *PtrICE1* were used for cold tolerance analysis. To assess cold tolerance, tobacco plants of different ages were subjected to cold treatment at either chilling (4 °C) or freezing (0 °C) temperature. The transgenic plants were morphologically indistinguishable from the WT under normal growth conditions. When 30-d-old seedlings were treated for 48h at 4 °C, the WT plants suffered more serious cold damage compared with the transgenic lines (Fig. 5A). Electrolyte leakage, a reliable indicator of cell membrane damage caused by abiotic stresses, was used to indicate the stress tolerance capacity. At the end of cold stress, electrolyte leakage of TG12 (9.8%) and TG22 (10.0%) plants was significantly lower than that of the WT (35.0%) (Fig. 5B). When the plants were exposed to cold stress (4 °C for 5 d), the transgenic lines displayed less serious damage in comparison with the WT (Fig. C). In another experiment, exposure of 35-d-old plants to 0 °C for 24h led to severe injury to the WT plants, whereas the transgenic plants were affected to a less serious degree (Fig. 5D). After recovery growth for 12 d in an ambient environment, the survival rate of WT plants was 24.7%, significantly lower than that of the transgenic lines: 88.2% for TG22 and 86.0% for TG12 (Fig. 5E). When larger plants were treated at 0 °C for 24h, the transgenic plants also displayed a better tolerance relative to the WT (Fig. 5F).

**Fig. 6.**
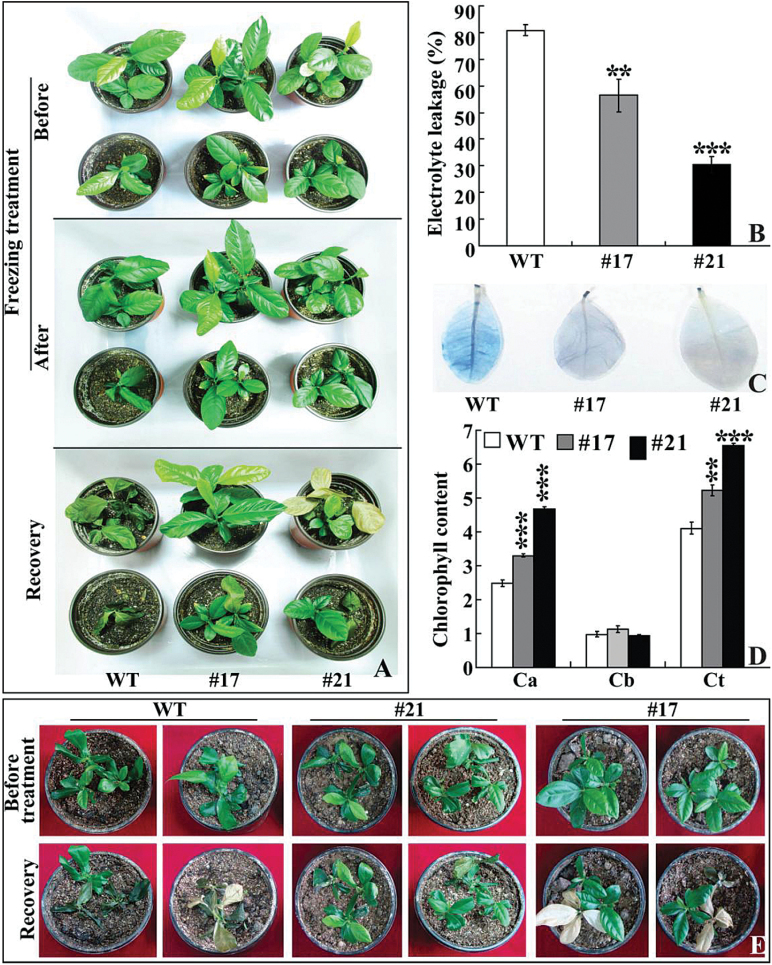
Overexpression of *PtrICE1* confers enhanced cold tolerance in lemon. (A) Plant phenotypes of lemon WT and transgenic lines (#17 and #21) before and after freezing treatment (–3 °C for 4h), followed by recovery growth for 5 d in an ambient environment. (B–D) Electrolyte leakage (B), cell death (C), and chlorophyll content (D) in WT and transgenic lines after freezing treatment. Asterisks indicate a significant difference between transgenic lines and WT (**P<0.01; ***P<0.001). Ca, chlorophyll *a*; Cb, chlorophyll *b*; Ct, total chlorophyll. (E) Tolerance assay of WT and transgenic lines subjected to freezing treatment (–6 °C for 1.5h), followed by 4 d recovery in an ambient environment. The upper panels are plants before treatment, while the bottom panels are plants after recovery. (This figure is available in colour at *JXB* online.)

PtrICE1 was also transferred into lemon to further characterize its role in cold tolerance. Two transgenic lemon lines (#17 and #21) exhibiting a higher abundance of *PtrICE1* mRNA, along with untransformed plants (WT), were subjected to cold treatment at freezing temperature. They were similar to each other in plant morphology in the absence of cold stress. Treatment at –3 °C for 4h led to conspicuous damage on the WT plants, but the damage on transgenic lines was less serious. After recovery growth for 5 d in an ambient environment, the WT plants failed to survive, whereas most of the transgenic plants grew healthily (Fig. 6A). WT had an electrolyte leakage of 80.9%, while electrolyte leakage of the two transgenic lines was significantly lower, 56.4% for #17 and 30.4% for #21 (Fig. 6B). Trypan blue staining was used to reveal cell death after the freezing treatment. As shown in Fig. 6C, the WT was more strongly stained in comparison with lines #17 and #21, suggesting that it experienced more detrimental damage. Chlorophyll level, a parameter for indicating leaf damage associated with cold stress, was also quantitatively measured. After the freezing treatment, chlorophyll *a* and total chlorophyll levels in the transgenic lines were considerably higher than those in the WT, but no significant difference in chlorophyll *b* was observed (Fig. 6D). These data indicated that overexpression of *PtrICE1* in both tobacco and lemon conferred enhanced cold tolerance of the transgenic plants.

### PtrICE1 interacts with PtADC

**Fig. 7.**
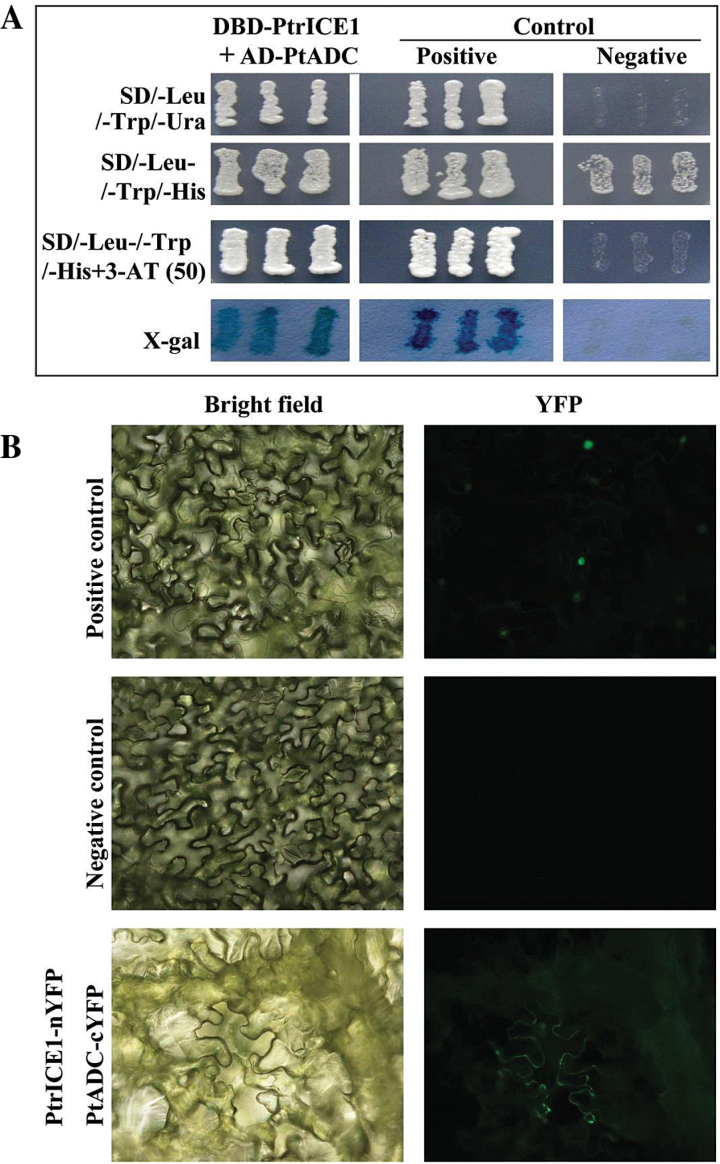
Analysis of the interaction between PtrICE1 and PtADC by Y2H and BiFC assays. (A) Growth of yeast cells of the positive control, negative control, and co-transformants of PtrICE1 and PtADC on SD/–Leu/–Trp/–Ura or SD/–Leu/–Trp/–His added with or without 3-AT. The blue colour indicates X-gal activity on SD/–Leu/–Trp medium. (B) BiFC assay using tobacco leaf epidermis. Representative images of the epidermal cells under bright field and fluorescence microscopy are shown. Positive and negative controls were bZIP63–cYFP+bZIP–nYFP and PtrICE1–nYFP+cYFP, respectively.

To elucidate the molecular mechanisms underlying the enhanced cold tolerance of transgenic plants overexpressing *PtrICE1*, a Y2H assay was carried out to identify proteins that may function in a complex with PtrICE1. Autoactivation was examined initially on the full-length or truncated PtrICE1; the assay showed that only full-length PtrICE1 could activate the reporter gene in the absence of prey (data not shown), suggesting that PtrICE1 could autoactivate itself. Therefore, truncated PtrICE1 was used to generate the bait for screening of a cDNA library. A total of 44 putative PtrICE1-interacting candidates were obtained. After sequencing, 21 positive proteins were identified (Table 1), due to the presence of more than one copy for some candidates. The PtrICE1-interacting proteins included transcription factors (bHLH122, MYB, and YABBY3), an abscisic acid receptor (PYR1), kinases (casein kinase and serine/threonine protein kinase), stress-associated proteins (arginine decarboxylase, Cu/Zn superoxide dismutase, and LEA5), and others. Interestingly, *PtADC* of trifoliate orange has been shown previously to play a role in cold tolerance (Wang *et al.*, 2011), which prompted us to focus on this protein. First, the interaction between PtrICE1 and PtADC was confirmed by a point-to-point Y2H assay. As shown in Fig. 7A, the yeast cells could grow on SD/-Leu/-Trp/-His medium. When 3-AT was added to the medium, only yeast cells of the positive control and co-transformant with bait and prey grew normally, whereas the negative control did not survive, consistent with the growth on SD/–Leu/–Trp/–Ura medium. The yeast growth was further supported by the assay of α-galactosidase activity. A BiFC assay was then performed to verify the Y2H analysis. Green fluorescence was observed in tobacco epidermis transformed with vectors containing PtrICE1–nYFP and PtADC–cYFP or with vectors of the positive control. In contrast, there was no fluorescence in the cells transformed with vectors of the negative control (Fig. 7B). Both the Y2H and BiFC assays indicated that PtrICE1 could interact with PtADC.

**Table 1.** Information about the PtrICE1-interacting proteins

No.	Accession no.	Details	Clones
1	Cs5g13930.1	21kDa seed protein; Miraculin	10
2	orange1.1t00168.2	40S ribosomal protein S3-3; 30S ribosomal protein S3P	2
3	Cs8g14970.1	Putative 60S ribosomal protein L10A;60S ribosomal protein L10a-3	1
4	orange1.1t01026.1	Abscisic acid receptor PYR1	1
5	Cs8g07560.2	Arginine decarboxylase	2
6	orange1.1t03173.1	Transcription factor bHLH122	1
7	Cs4g16350.1	Casein kinase I isoform delta-like; casein kinase I isoform delta-A	2
8	Cs3g27290.1	Catalase	3
9	Cs3g12080.1	Cu/Zn superoxide dismutase	1
10	Cs5g07190	Elongation factor 1-delta 2	3
11	Cs4g18520.1	Glycine dehydrogenase A	2
12	Cs2g20230.2	Glycine-rich protein	1
13	Cs9g04210.1	Late embryogenesis abundant protein Lea5	2
14	Cs1g24390	Mannan endo-1,4-β- mannosidase 7	1
15	orange1.1t05358.1	Putative enoyl-CoA hydratase/isomerase yngF	2
16	Cs2g06140.2	Monothiol glutaredoxin-S17; glutaredoxin-3	1
17	Cs5g04740.1	Transcription factor MYB1R1	1
18	Cs1g21570.2	NADH dehydrogenase [ubiquinone] iron-sulfur protein 4	1
19	Cs1g21570.2	NADH-ubiquinone oxidoreductase	4
20	Cs4g04760.1	Serine/threonine protein kinase	1
21	Cs7g19090.1	Tubulin-binding cofactor C domain-containing protein 1	2

### Transgenic plants have higher levels of *ADC* transcripts and free polyamines

**Fig. 8.**
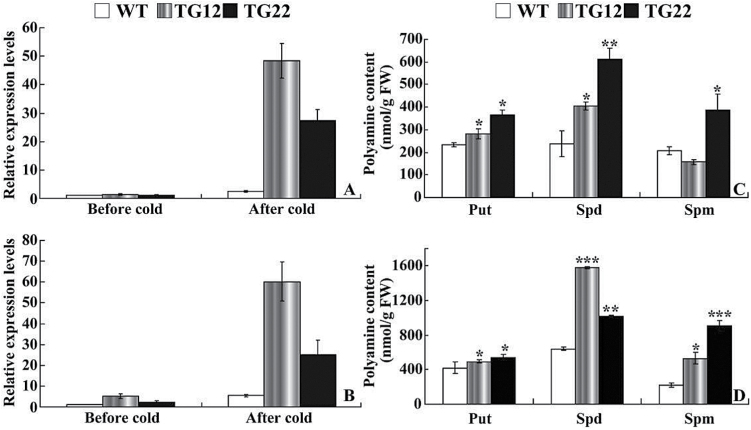
Analysis of *ADC* expression and free polyamines in tobacco. (A, B) Expression patterns of *NtADC1* (A) and *NtADC2* (B) in tobacco WT and transgenic lines before and after cold stress. (C, D) Free polyamine contents in WT and transgenic lines before (C) and after (D) cold stress. Put, putrescine; Spd, spermidine; Spm, spermine. Asterisks indicate a significant difference between transgenic lines and the WT at the same time point (*P<0.05; **P<0.01; ***P<0.001).

ADC is a key enzyme responsible for the synthesis of putrescine, which is then converted into spermidine and spermine. Efforts were thus made to compare the levels of *ADC* transcripts and free polyamines between transgenic lines and the WT. Under non-stressful conditions, mRNA levels of *NtADC1* and *NtADC2* in the transgenic tobacco lines (TG12 and TG22) were slightly higher than those of the WT. Exposure to cold stress caused minor upregulation of *NtADC1* and *NtADC2* in the WT, but greater induction was observed in the transgenic lines. The transcript levels of *NtADC1* in TG12 and TG22 were 19.8 and 11.2 times that in the WT, respectively, while *NtADC2* levels in the two transgenic lines were 4.1–11.1 times that in the WT (Fig. 8A, B).

HPLC measurement showed that, in the absence of cold stress, the putrescine content of the WT was 233.5 nmol per g of fresh weight, while TG12 and TG22 had putrescine levels of 282.3 and 363.4 nmol per g of fresh weight, respectively (Fig. 8C). The contents of spermidine and spermine in the two transgenic lines were significantly higher than those of the WT except spermine of TG12. Cold treatment elevated the three polyamines in all tested lines, but it was noticeable that the polyamine levels in TG12 and TG22 were higher than those of the WT (Fig. 8D).

**Fig. 9.**
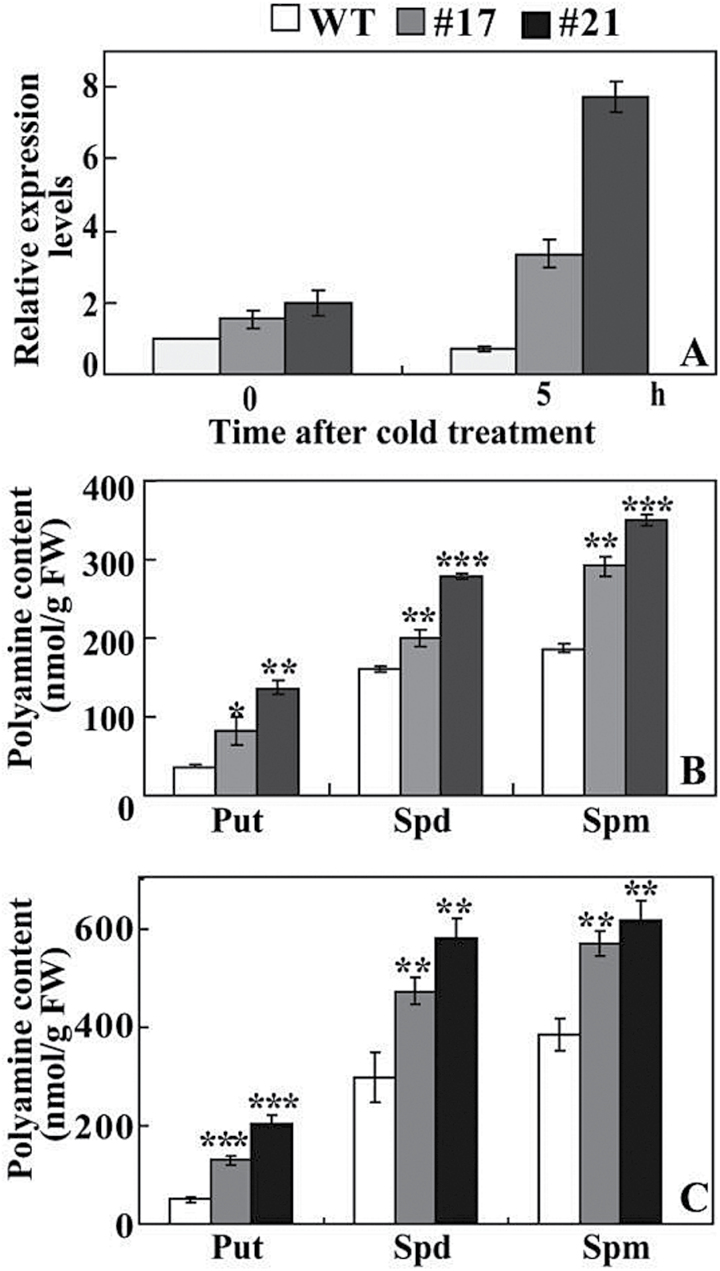
Analysis of *ADC* expression and free polyamines in lemon. (A) Expression patterns of *ClADC* in lemon WT and transgenic lines before and after 5h of cold stress. (B) Free polyamine contents in WT and transgenic lines before and after cold stress. Put, putrescine; Spd, spermidine; Spm, spermine. Asterisks indicate a significant difference between transgenic lines and the WT at the same time point (*P<0.05; **P<0.01; ***P<0.001).

Expression levels of *ClADC* in lemon were increased in the two transgenic lines when compared with the WT without cold stress, although the difference was not dramatic. After cold treatment for 5h, *ClADC* transcripts of #17 and #21 were remarkably enhanced, in contrast to a slight induction in the WT (Fig. 9A). The polyamine contents of the two transgenic lines were significantly higher than those of the WT without cold stress (Fig. 9B). Cold treatment increased the levels of putrescine, spermidine, and spermine, whereas the elevation in the transgenic lines was more substantial than in the WT. As a result, #17 and #21 displayed higher levels of the three types of polyamines than the WT did (Fig. 9C).

### Transgenic lines accumulate less ROS and exhibit higher antioxidant enzyme activities under cold stress

**Fig. 10.**
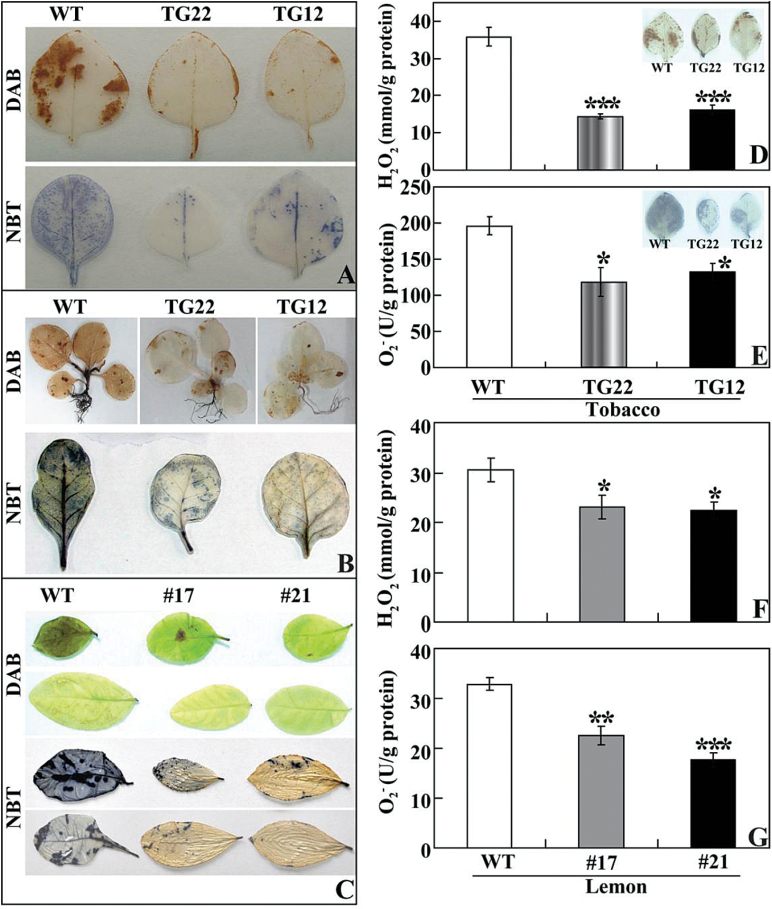
Analysis of H_2_O_2_ and O_2_^–^ in tobacco and lemon under cold stress. (A, B) Representative images indicating *in situ* accumulation of H_2_O_2_ and O_2_^–^ in tobacco WT and transgenic lines (TG22 and TG12) after cold stress at chilling (A) and freezing (B) temperatures. (C) Representative images indicating *in situ* accumulation of H_2_O_2_ and O_2_^–^ in lemon WT and transgenic lines (#17 and #21) after cold stress. (D, E) Levels of H_2_O_2_ (D) and O_2_^–^ (E) in tobacco WT and transgenic lines (TG22 and TG12) after cold treatment. The insets in (D) and (E) are histochemical staining patterns using DAB and NBT, respectively. (F, G) Levels of H_2_O_2_ (F) and O_2_^–^ (G) in lemon WT and transgenic lines (#17 and #21) after cold stress at freezing temperatures. Asterisks indicate significant difference between transgenic lines and the WT (*P<0.05; **P<0.01; ***P<0.001).

It has been suggested that polyamines are involved in the modulation of ROS homeostasis under abiotic stresses (Liu *et al.*, 2007; Moschou *et al.*, 2008; Tiburcio *et al.*, 2014), so we determined ROS levels in the tested samples. We first examined *in situ* accumulation of H_2_O_2_ and O_2_^–^ by histochemical staining with DAB and NBT, respectively. After cold stress at chilling or freezing temperatures, the leaves of transgenic tobacco (Fig. 10A, B) and lemon (Fig. 10C) were stained to a lighter extent compared with those of the corresponding WT. Quantitative measurement further demonstrated that H_2_O_2_ and O_2_^–^ contents in the transgenic lines of tobacco (Fig. 10D, E) and lemon (Fig. 10F, G) were lower than in the WT. Both histochemical staining and quantitative measurement indicated that the transgenic lines accumulated lower levels of ROS under the cold stresses.

**Fig. 11.**
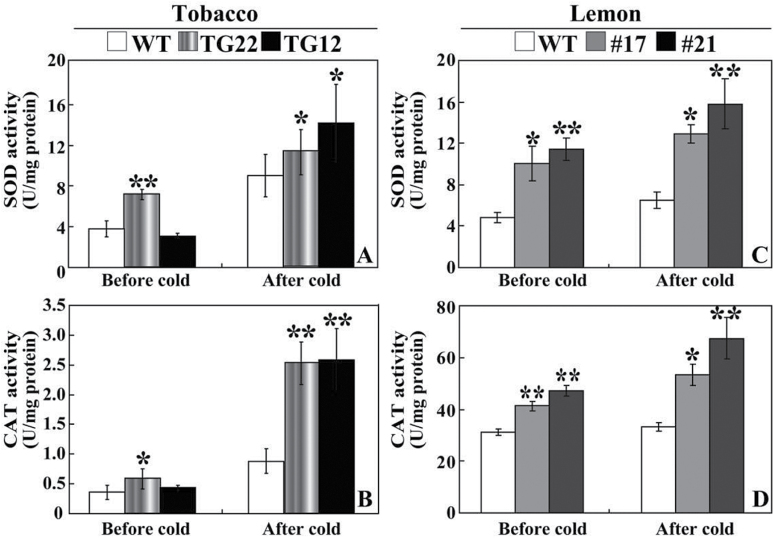
Analysis of SOD and CAT activities in tobacco and lemon. (A, B) Activity of SOD (A) and CAT (B) in tobacco WT and transgenic lines (TG22 and TG12) before and after cold treatment. (C, D) Activity of SOD (C) and CAT (D) in lemon WT and transgenic lines (#17 and #21) before and after cold stress. Asterisks indicate a significant difference between transgenic lines and the WT (*P<0.05; **P<0.01).

The fact that antioxidant enzymes, such as SOD and catalase (CAT), play crucial roles in ROS detoxification (Gill and Tuteja, 2010) and that they were isolated by Y2H (Table 1) prompted us to assess the enzyme activities of SOD and CAT. Without cold tolerance, the enzyme activities of SOD and CAT in tobacco transgenic lines were higher than (TG22) or similar to (TG12) to those of the WT. Exposure to cold increased the activities of both enzymes, but the activities of the transgenic lines were significantly higher than in the WT (Fig. 11 A, B). Likewise, the enzyme activities of SOD and CAT in the two lemon transgenic lines (#17 and #21) were significantly higher than those of the WT before and after cold treatment (Fig. 11 C, D).

## Discussion

bHLH proteins constitute one of the largest transcription factor families in plants; 177 and 167 bHLHs have been unravelled in the genomes of rice and *Arabidopsis thaliana* (Bailey *et al.*, 2003; Toledo-Ortiz *et al.*, 2003; Li *et al.*, 2006; Carretero-Paulet *et al.*, 2010). Recently, 117 transcript models encoding canonical bHLHs were identified in the lotus genome (Hudson and Hudson, 2014). Numerous studies indicate that plant bHLH proteins are tightly implicated in a variety of biological processes, such as cell specification (Bernhardt *et al.*, 2003), cell elongation (Bai *et al.*, 2012), flowering (Ito *et al.*, 2012), pollen development (Ko *et al.*, 2014), hormone signalling response (Sasaki-Sekimoto *et al.*, 2014), and secondary metabolism (Xie *et al.*, 2012; Ji *et al.*, 2014). In addition, accumulating evidence shows that bHLHs play pivotal roles in plant responses to various abiotic stresses, including iron deficiency (Sivitz *et al.*, 2012), drought (W. Liu *et al.*, 2014), and cold (Chinnusamy *et al.*, 2003; Chen *et al.*, 2013; Huang *et al.*, 2013; Peng *et al.*, 2014). ICE1 is a well-characterized bHLH protein that acts as an upstream regulator of the transcriptional regulation cascade of the cold response in *Arabidopsis* (Toledo-Ortiz *et al.*, 2003). However, little is known about the roles of ICE1 homologues in trifoliate orange, a very cold-hardy plant. Thus, characterization of an *ICE1* gene of trifoliate orange is crucial to decipher the cold signalling pathway pertinent to freezing tolerance and to provide valuable gene candidates for genetic manipulation.

The bHLH proteins are defined by the presence of conserved bHLH signature domains consisting of two distinct segments, a basic region at the N-terminal end and an HLH region at the C-terminal end (Buck and Atchley, 2003; Toledo-Ortiz *et al.*, 2003; Li *et al.*, 2006). The HLH region contains two amphipathic helices, composed predominantly of hydrophobic residues, which are separated by a loop region of variable length. The basic region, typically rich in basic amino acids, has been shown to be critical for DNA binding, while the HLH domain is responsible for protein–protein interactions to form either homo- or heterodimeric complexes (Toledo-Ortiz *et al.*, 2003). As well as these key signatures, bHLHs contain a leucine-zipper motif characterized by heptad repeats of leucine residues, adjacent to the second helix in the bHLH region (Hudson and Hudson, 2014). In this study, PtrICE1 has the entire set of above-mentioned signature motifs required for defining a typical bHLH transcription factor, despite a low degree of sequence conservation outside the bHLH domain. According to the criterion proposed by Toledo-Ortiz *et al.* (2003), PtrICE1 should be classified into the category of E-box binders as it contains two specific residues, glutamate (E) and arginine (R), in the basic region. This was supported by a yeast one-hybrid assay, in which PtrICE1 was revealed to recognize a DNA sequence harbouring the E-box element. Multiple alignments revealed that PtrICE1 shared a striking sequence similarity with AtICE1 of *Arabidopsis* and other plants, indicating that PtrICE1 is a putative ICE1 homologue of trifoliate orange.

*PtrICE1* was induced by abiotic stresses, including cold, salt, and dehydration. Upregulation of *PtrICE1* by salt and cold was consistent with the expression profiles of *AtICE1*, but they differed in the response to dehydration, as *AtICE1* was not induced under dehydration (Chinnusamy *et al.*, 2003). The disparity of expression patterns between *PtrICE1* and *AtICE1* in response to dehydration might be presumably ascribed to the inherent difference in plant species. However, durations of dehydration treatment in these studies may also account for the difference, as shorter time frame (30min) was used for *Arabidopsis thaliana* in comparison with our work. The strongest induction of *PtrICE1* transcripts by cold stress forced us to elucidate its function in cold tolerance. The assays demonstrated that overexpression of *PtrICE1* in either annual (tobacco) or perennial (lemon) plants resulted in pronouncedly enhanced tolerance to cold stresses, indicating that PtrICE1 acts as a positive regulator of cold signalling cascade. Meanwhile, overexpression of *PtrICE1* did not cause negative impacts on plant growth of the transgenic lines under normal growth conditions, suggesting that *PtrICE1* might hold great potential for genetic engineering to improve cold tolerance.

*ICE1* plays a critical role in cold response by positively regulating *CBF3* through binding specifically to the MYCR element in the promoter region (Chinnusamy *et al.*, 2003). This regulation is considered as a classical mode of action on *ICE1*, which is also reasonable as *ICE1*, encoding a bHLH transcription factor, might function in cold signalling via transcriptional regulation of its target genes. However, it is worth mentioning that, as protein–protein interactions are important for executing gene function, exploration of PtrICE1-interacting protein may shed new light on the mechanisms underlying enhanced cold tolerance from a different aspect. As a matter of fact, bHLH proteins have been revealed to interact with other non-bHLH transcription factors or functional proteins, forming protein complexes, to participate in various cellular processes. For example, an interaction between MYBs and bHLHs has been shown to play an essential role in governing the synthesis of secondary metabolites, such as glucosinolate and anthocyanin (Xie *et al.*, 2012; Frerigmann and Gigolashvili, 2014). In another work, a dehydrin protein of *Medicago truncatula*, MtCAS (cold-acclimation specific protein 31), was reported to interact with MtICE1 and influenced stomatal development (Xie *et al.*, 2012). In the current study, Y2H analysis revealed that a total of 21 proteins might constitute the PtrICE1 interactome, among which two proteins (MYB1R1 and bHLH122) belonged to MYB and bHLH families that are capable of interacting with bHLH protein (Xie *et al.*, 2012; Ko *et al.*, 2014). In the future, more work is required to experimentally clarify the interaction between these two proteins and PtrICE1, and to decipher their role in cold tolerance. Interestingly, the interactome included several functional proteins associated with stress tolerance, such as SOD, CAT, late embryogenesis abundant protein (LEA), and ADC. These proteins function, directly or indirectly, in counteracting the abiotic stresses. For instance, SOD and CAT are important antioxidant enzymes for scavenging ROS produced under abiotic stresses (McKersie *et al.*, 1993; Chen *et al.*, 2013). LEAs are hydrophilic proteins involved in the stabilization of proteins and membrane, and the protection of enzymes (Olvera-Carrillo *et al.*, 2011). ADC is a key enzyme responsible for synthesis of polyamines that has been suggested to play a protective role in combating abiotic stresses (Shi and Chan, 2013; Tiburcio *et al.*, 2014). Based on these illustrations, it is tempting to hypothesize that the interaction between PtrICE1 and the stress-associated proteins may work in concert with the well-defined ICE1–CBF3 signalling pathway to fight against cold stress.

PtADC was validated as the *bona fide* protein interacting with PtrICE1, which led us to examine the expression of *ADC* genes and endogenous polyamine levels in the transgenic plants. In the absence of cold stress, expression levels of *ADC* genes in tobacco and lemon transgenic lines were only slightly higher than in the WT. However, exposure to the cold stress resulted in more pronounced induction of the *ADC* genes in the transgenic lines compared with the WT, leading to more noticeable differences in the transcript levels between them. Whether the interaction accounts for this increase remains to be investigated. Changes in the *ADC* expression patterns agreed with that of *CBF3*, which was not strongly activated at warm temperatures but was dramatically induced by cold, in the transgenic lines overexpressing *ICE1* (Chinnusamy *et al.*, 2003). It is now clear that post-transcriptional modification, such as sumoylation and ubiquitination (Dong *et al.*, 2006; Miura *et al.*, 2007, 2011), influence the regulation of ICE1 on CBF3 under cold conditions. However, it remains to be investigated whether greater activation of *ADC* genes in the transgenic lines results from cold-induced modification of PtrICE1. The greater abundance of *ADC* mRNA was concurrent with higher putrescine levels in the transgenic lines in comparison with the WT. This, in turn, was accompanied by significantly higher levels of spermidine and spermine in the transgenic lines, except for spermine levels in TG12 before cold stress. As putrescine is the precursor of spermidine and spermine, active synthesis of putrescine may cause a feed-forward stimulation of downstream metabolism, leading to the promotion of spermidine and spermine synthesis. It was noticed that higher polyamine levels in the transgenic lines were consistent with enhanced cold tolerance, whereas pre-treatment of the tobacco transgenic lines with d-arginine, an ADC inhibitor, drastically compromised the cold tolerance (data not shown). These results suggest that elevation of polyamine synthesis might contribute to the improvement of cold tolerance in the transgenic lines overexpressing *PtrICE1*. Our data corroborate previous studies indicating the important roles of polyamines in cold stress tolerance (Shen *et al.*, 2000; Alcázar *et al.*, 2011). In an earlier work, accumulation of putrescine was essential for cold acclimation and plant survival at freezing temperature in *Arabidopsis thaliana* (Cuevas *et al.*, 2008). In addition, an increase in endogenous polyamine levels by overexpression of polyamine biosynthetic genes or exogenous application of polyamines has been demonstrated to enhance cold tolerance in various plants (Wang *et al.*, 2011; Xu *et al.*, 2011; Song *et al.*, 2014). Polyamines have been suggested to function in elevating stress tolerance due to maintenance of ROS homeostasis by modulating the antioxidant system (Liu *et al.*, 2007; Alcázar *et al.*, 2010; Shi *et al.*, 2010; Wang *et al.*, 2011; Shi and Chan, 2014; Song *et al.*, 2014). Therefore, elevation of the polyamine pool may, along with activated antioxidant enzymes like SOD and CAT, provide a robust ROS detoxification system in the transgenic lines. As a consequence, ROS produced in these lines under cold stress may be detoxified in a more efficient manner, leading to alleviation of oxidative stresses and cold damage. This notion is supported, at least in part, by accumulation of less ROS in the transgenic lines relative to the WT under cold stress conditions.

Taken together, *PtrICE1* of trifoliate orange was upregulated by various abiotic stresses, including cold, salt, and dehydration, with the strongest induction under cold conditions. Overexpression of *PtrICE1* in tobacco and lemon conferred enhanced tolerance to cold at either chilling or freezing temperatures. A total of 21 proteins were identified as potential candidates interacting with PtrICE1, among which PtADC was confirmed as a true member in the interactome. In addition, higher levels of *ADC* transcripts and free polyamines were detected in the transgenic lines, concomitant with accumulation of less ROS. All of these results demonstrate that *PtrICE1* functions positively in cold tolerance by promoting polyamine accumulation by interacting with ADC. The current study provides new knowledge of the function and underlying mechanism of *ICE1* and expands our understanding of the complex cold signalling network.

## Supplementary data

Supplementary data are available at *JXB* online.

Supplementary Fig. S1. Phylogenetic comparison of PtrICE1 (shown by a red circle) and 16 ICE1 proteins of other plants.

Supplementary Fig. S2. Alignment between PtrICE1 and PtrbHLH, an ICE1-like protein.

Supplementary Table S1. Primers used in this study.

Supplementary Data

## Abbreviations:

AD: activation domain
BD: DNA-binding domain
BiFC: bimolecular fluorescence complementation
bHLH: basic helix–loop–helix
CaMV: cauliflower mosaic virus
DAB: 3,3’-diaminobenzidine
EST: expressed sequence tag
HPLC: high-performance liquid chromatography
NBT: nitroblue tetrazolium
ORF: open reading frame
qPCR: quantitative real-time RT-PCR
RACE: rapid amplification of cDNA ends
ROS: reactive oxygen species
RT-PCR: reverse transcription-PCR
Y2H: yeast two-hybrid
YFP: yellow fluorescent protein.
