# Supplementary material for: ICE1 of Poncirus trifoliata functions in cold tolerance by modulating polyamine levels through interacting with arginine decarboxylase
Source: J Exp Bot. 2015 Apr 6;66(11):3259–74. doi: 10.1093/jxb/erv138 (PMC4449543; doi:10.1093/jxb/erv138)
Supplement: Supplementary Data [file supp_66_11_3259__index.html]

 ICE1 of Poncirus trifoliata functions in cold tolerance by modulating polyamine levels through interacting with arginine decarboxylase — ICE1 of Poncirus trifoliata functions in cold tolerance by modulating polyamine levels through interacting with arginine decarboxylase — Supplementary Data 

# *ICE1* of *Poncirus trifoliata* functions in cold tolerance by modulating polyamine levels through interacting with arginine decarboxylase

## Supplementary Data

Data files

**Files in this Data Supplement:**

- Supplementary Data - Supplementary Data
