# Supplementary material for: ICE1 of Poncirus trifoliata functions in cold tolerance by modulating polyamine levels through interacting with arginine decarboxylase
Source: J Exp Bot. 2015 Apr 6;66(11):3259–74. doi: 10.1093/jxb/erv138 (PMC4449543; doi:10.1093/jxb/erv138)
Supplement: Supplementary Data [file supp_erv138_jexbot144717_file001.pdf]

ICE1 of Poncirus trifoliata functions in cold tolerance by modulating polyamine levels through interacting with arginine decarboxylase

Xiao-San Huang, Qinghua Zhang, Dexin Zhu, Xingzheng Fu, Min Wang, Qian Zhang, Takaya Moriguchi, Ji-Hong Liu

Supplementary data

Figure legends

Figure S1. Phylogenetic comparison of PtrICE1 (shown by a red circle) and 16 ICE1 proteins of other plants. Accession numbers of the 16 ICE1 proteins used are as follows. AtICE1(AAP14668); AtICE2(BAC42644); CbICE1(AAS79350); PtICE1(ABN58427); PsICE1(ABF48720); MdICE1(ABS50251); GmICE1(ACJ39211); GmICE2(ACJ39212); GmICE3(ACJ39213); GmICE4(ACJ39214); RcICE1(EEF51703); OsICE1(BAD88163); TaICE41(ACB69501); TaICE87(ACB69502); ZmICE2(ACG46593); OrbHLH2(ABA93991);

Figure S2. Alignment between PtrICE1 and PtrbHLH, an ICE1-like protein.

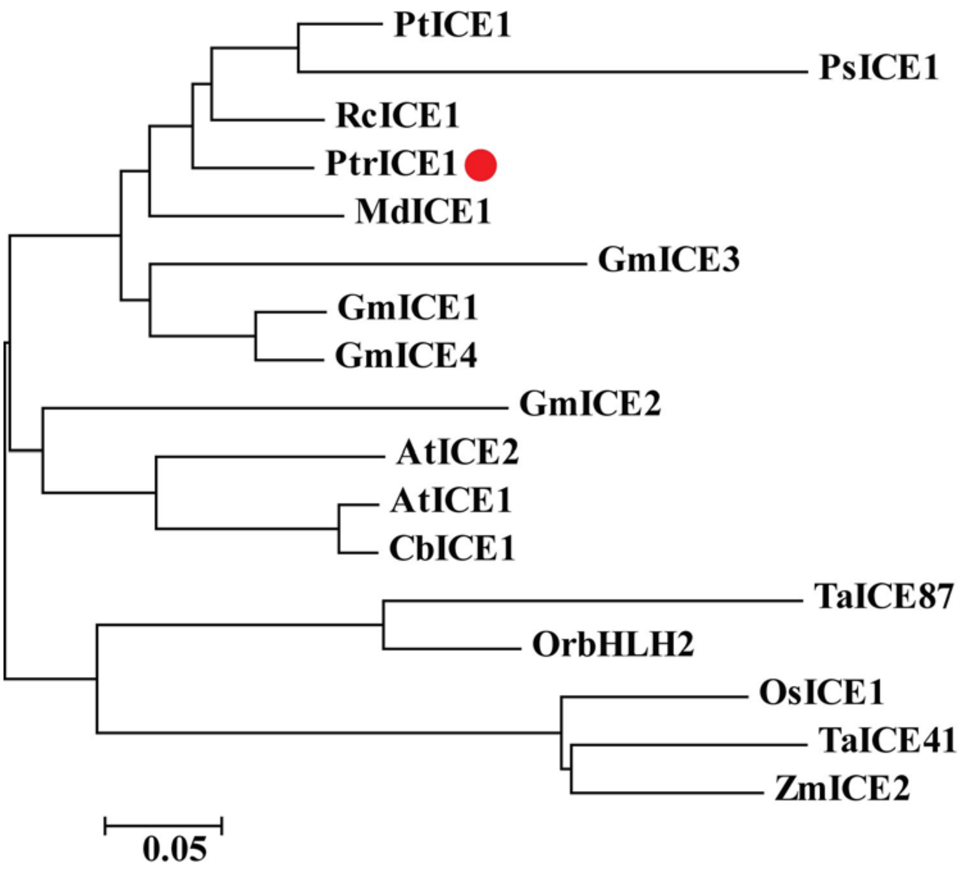
PtICE1
PsICE1
RcICE1
PtrICE1
MdICE1
GmICE3
GmICE1
GmICE4
GmICE2
AtICE2
AtICE1
CbICE1
TaICE87
OrbHLH2
OsICE1
TaICE41
ZmICE2
0.05

Figure S1.

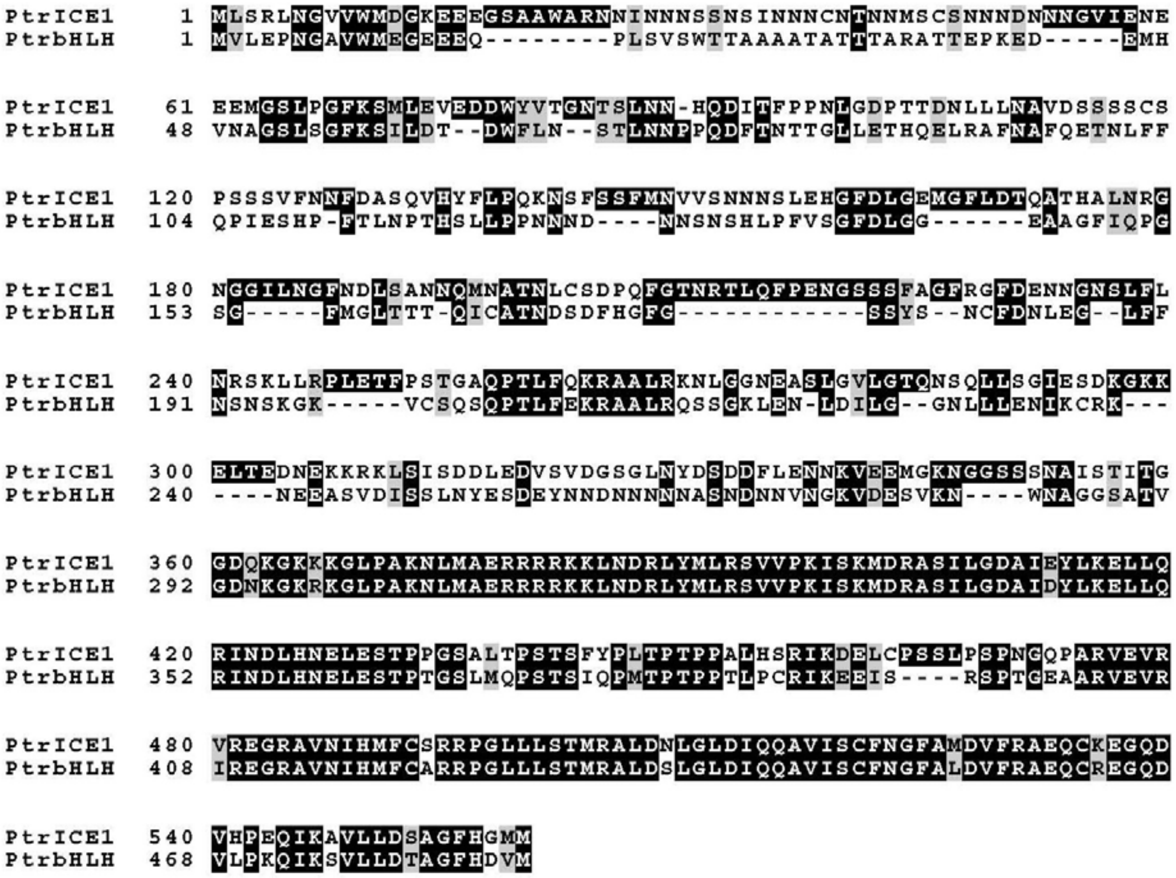
PtrICE1 1 MLSRLNGVVWMDGKEEEGSAAWARNNINNNSSNSINNNCNTNNMSCSNNNDNNNGVIENE
PtrbHLH 1 MVLEPNGAVWMEGEEEQ--------PLSVSWTTAAAATATTTARATTEPKED-----EMH
PtrICE1 61 EEMGSLPGFKSMLEVEDDWYVTGNTSLNN-HQDITFPPNLGDPTTDNLLLNAVDSSSSCS
PtrbHLH 48 VNAGSLSGFKSILDT--DWFLN--STLNNPPQDFTNTTGLLETHQELRAFNAFQETNLFF
PtrICE1 120 PSSSVFNNFDASQVHYFLPQKNSFSSFMNVVSNNNSLEHGFDLGEMGFLDTQATHALNRG
PtrbHLH 104 QPIESHP-FTLNPTHSLLPPNNND----NNSNSHLPFVSGFDLGG------EAAGFIQPG
PtrICE1 180 NGGILNGFNDLSANNQMNATNLCSDPQFGTNRTLQFPENGSSSFAGFRGFDENNGNSLFL
PtrbHLH 153 SG-----FMGLTTT-QICATNDSDFHGFG------------SSYS--NCFDNLEG--LFF
PtrICE1 240 NRSKLLRPLETFPSTGAQPTLFQKRAALRKNLGGNEASLGVLGTQNSQLLSGIESDKGKK
PtrbHLH 191 NSNSKGK-----VCSQSQPTLFEKRAALRQSSGKLEN-LDILG--GNLLLENIKCRK---
PtrICE1 300 ELTEDNEKKRKLSISDDLEDVSVDGSGLNYDSDDFLENNKVEEMGKNGGSSSNAISTITG
PtrbHLH 240 ----NEEASVDISSLNYESDEYNNDNNNNNASNDNNVNGKVDESVKN----WNAGGSATV
PtrICE1 360 GDQKGKKKGLPAKNLMAERRRRKKLNDRLYMLRSVVPKISKMDRASILGDAIEYLKELLQ
PtrbHLH 292 GDNKGKRKGLPAKNLMAERRRRKKLNDRLYMLRSVVPKISKMDRASILGDAIDYLKELLQ
PtrICE1 420 RINDLHNELESTPPGSALTPSTSFYPLTPTPPALHSRIKDELCPSSLPSPNGQPARVEVR
PtrbHLH 352 RINDLHNELESTPTGSLMQPSTSIQPMTPTPPTLPCRIKEEIS----RSPTGEAARVEVR
PtrICE1 480 VREGRAVNIHMFCSRRPGLLLSTMRALDNLGLDIQQAVISCFNGFAMDVFRAEQCKEGQD
PtrbHLH 408 IREGRAVNIHMFCARRPGLLLSTMRALDSLGLDIQQAVISCFNGFALDVFRAEQCREGQD
PtrICE1 540 VHPEQIKAVLLDSAGFHGMM
PtrbHLH 468 VLPKQIKSVLLDTAGFHDVM

Figure S2.

Table S1. Primers used in this study.

Purposes	Primers	Sequences (5'-3')	
		Forward	Reverse
**5'-RACE PCR**	GSP1	TTGTCGACCTCTCTGCATCTGCTGAGCTGCTG	ATGGTACCGCCCTTCGAGGAGCCGAACATTGT
**Subcellular localization**	GSP2	ATCTCGAGATGCTTTCTAGACTAAACGGTGTGG	TACCATGGCATCATGCCATGGAAGCCGGCTGA
**Transcriptional activation**	GSP3	TACCATGGATGCTTTCTAGACTAAACGGTGTGG	GGCTCGAGCATCATGCCATGGAAGCCGGCTGA
**Transcriptional binding**	GSP4	ATCCATGGTGATGCTTTCTAGACTAAACGGTGTGG	GGCTCGAGCATCATGCCATGGAAGCCGGCTGA
**Transgenic confirmation**	NPTII	AGACAATCGGCTGCTCTGAT	TCATTTCGAACCCCAGAGTC
	CaMV 35S-PtrICE1	CGCCGTAAAGACTGGCGAACAGTTCATACAGAGT	GCCCTTCGAGGAGCCGAACATTGT
**Transgene overexpression**	GSP5	CCTGGATTCAAGTCCATGCT	CCAAGGTTTGGTGGAAATGT
**Y2H screening**	GSP6	CGCCGTAAAGACTGGCGAACAGTTCATACAGAGT	ATGGTACCACAATGTTCGGCTCCTCGAAGGGC
**Y2H**	GSP7	CGGGGTACCCCCCCTCGTTTTTTCTTTTTCTT	CCGCTCGAGTGTTCAACTGCTTCCATCTTTTG
	GSP8	CGGGGTACCCTTGGTGACCCAACAACTGA	CCGCTCGAGATGCCATGGAAGCCGGCTGAAT
**BiFC**	GSP9	CGCCATGGATGCTTTCTAGACTAAACGGTGTG	CCATCGATCATCATGCCATGGAAGCCG
	GSP10	GACTAGTATGCCGGCCCTCGGGTGTT	GGGGTACCAGCATAGCAGTATGACCACTGCTC
	bZIP63-BiFC	CGGGATCCATGGAAAAAGTTTTCTCCGACG	CCGCTCGAGCTGATCCCCAACGCTTCGAAT
**Gene expression**	ClADC	AGTCATTGACATCGGGGGCG	GCTTGAACAACGGCAGAGGC
	NtADC1	CTTGCTGATTACCGCAATTTATC	CCTTACTGCAGGCTTTTCATCTA
	NtADC2	GCCGGCCCTAGGTTGTTGTGTAGATG	AGCGAACAACAAGAGGCAGCTGAAGCC
	Actin	CATCCCTCAGCACCTTCC	CCAACCTTAGCACTTCTCC
	NtUbiquitin	TCCAGGACAAGGAGGGTAT	CATCAACAACAGGCAACCTAG
